# Anticancer Potential of L-Histidine-Capped Silver Nanoparticles against Human Cervical Cancer Cells (SiHA)

**DOI:** 10.3390/nano11113154

**Published:** 2021-11-22

**Authors:** Rajmohamed Mohammed Asik, Chidhambaram Manikkaraja, Karuppusamy Tamil Surya, Natarajan Suganthy, Archunan Priya Aarthy, Domokos Mathe, Muthusamy Sivakumar, Govindaraju Archunan, Parasuraman Padmanabhan, Balazs Gulyas

**Affiliations:** 1 Department of Animal Science, Bharathidasan University, Tiruchirappalli 620024, India; mdasik91@gmail.com (R.M.A.); mraja.vishnu@gmail.com (C.M.); suriyakalyaniyadav@gmail.com (K.T.S.); 2 Department of Nanoscience and Technology, Alagappa University, Karaikudi 630003, India; suganthy.n@gmail.com; 3 Department of Obstetrics and Gynecology, Rabindra Nath Tagore Medical College, Udaipur 313001, India; aarthyarchun94@gmail.com; 4 Department of Biophysics and Radiation Biology, Semmelweis University, 1094 Budapest, Hungary; domokos.mathe@hcemm.eu; 5 CROmed Translational Research Centers Ltd., 1094 Budapest, Hungary; 6 In Vivo Imaging Advanced Core Facility, Hungarian Center of Excellence for Molecular Medicine (HCEMM), 1094 Budapest, Hungary; 7 Ariviya Technologies Pvt Ltd., Pattukkottai, Thanjavur 614602, India; drsiva@ariviya.com; 8 Dean-Research, Marudupandiyar College, Thanjavur 613403, India; 9 Lee Kong Chian School of Medicine, Nanyang Technological University, Singapore 636921, Singapore; balazs.gulyas@ntu.edu.sg; 10 Cognitive Neuroimaging Centre, Nanyang Technological University, 59 Nanyang Drive, Singapore 636921, Singapore; 11 Department of Clinical Neuroscience, Karolinska Institute, 17176 Stockholm, Sweden

**Keywords:** anticancer activity, L-histidine, silver nanoparticles, cervical cancer cells, apoptosis

## Abstract

This study reports the synthesis of silver nanoparticles using amino acid L-histidine as a reducing and capping agent as an eco-friendly approach. Fabricated L-histidine-capped silver nanoparticles (L-HAgNPs) were characterized by spectroscopic and microscopic studies. Spherical shaped L-HAgNPs were synthesized with a particle size of 47.43 ± 19.83 nm and zeta potential of −20.5 ± 0.95 mV. Results of the anticancer potential of L-HAgNPs showed antiproliferative effect against SiHa cells in a dose-dependent manner with an IC_50_ value of 18.25 ± 0.36 µg/mL. Fluorescent microscopic analysis revealed L-HAgNPs induced reactive oxygen species (ROS) mediated mitochondrial dysfunction, leading to activation of apoptotic pathway and DNA damage eventually causing cell death. To conclude, L-HAgNPs can act as promising candidates for cervical cancer therapy.

## 1. Introduction

Nanotechnology is the engineering of functional systems at the molecular scale. In recent years, metal nanoparticles have become powerful materials in biomedicine [1,2,3]. Unique physicochemical properties of metal nanoparticles have increased the attention of researchers in recent decades in various fields such as catalysis and optoelectronics [4]. Among the metal nanoparticles, silver nanoparticles (AgNPs) have received much attention in the field of biomedicine due to their wide pharmacological properties such as antibacterial, antifungal, anti-inflammatory, antiviral activities, and biosensing properties [5,6,7,8,9]. Conventionally silver nanoparticles were synthesized through physical and chemical methods, which suffered certain limitations provoking the researchers toward biogenic sources for the synthesis of nanoparticles [10]. Multiple lines of evidence revealed the green synthesis of silver nanoparticles via plant, microbial, and algal sources [11]. Among the bioactive compounds from natural sources, amino acids have been used recently as biogenic sources, in which the amino, carboxyl, and side-chain groups act as perfect reducing and capping agents, playing vital roles in the morphology of synthesized nanoparticles. In addition, amino-acid-capped nanoparticles are biocompatible in nature, with good mechanical and thermal properties [12]. Natural amino acids such as glycine, tyrosine, and tryptophan have been used as reducing and surface functionalization agents for the fabrication of Ag nanoparticles coated thin films, which reported potent antibacterial and antibiofilm activities [13]. The amino acid L-histidine used in this study has an aliphatic amino group (pKa around 9.4) on the side chain and an imidazole group with a pKa ≈ 6.04, which becomes protonated at acidic pH. Few studies have been reported on L-histidine-based nanoparticle synthesis for biomedical applications [14,15]. This study focused on an eco-friendly approach for the synthesis of L-histidine-capped silver nanoparticles (L-HAgNPs). Cervical cancer is the second-most prevalent cause of cancer-induced mortality in women mostly in developed countries (90%), with a death rate of 3.5 million in 2012, which might increase to 5.5 million in 2030 if no effective therapy intervenes. Clinical and epidemiological survey reveals that people with human papilloma viral infection (HPV) have a high risk of cervical cancer. Treatment strategies available for cervical cancer include electrosurgical excision procedure, surgery, radiation, and chemotherapy, or a combination of chemo and radiation therapy, which posed severe side effects affecting the immune system, thereby enhancing the host susceptibility to disease, multidrug resistance, and toxicity [16]. Recent research revealed that nanoparticle-based therapy showed enhanced anticancer potential with reduced side effects. Multiple lines of evidence illustrated that noble metal nanoparticles synthesized through plant sources showed promising anticancer potential against cervical cancer in conjugation with chemotherapeutic drugs [17,18,19]. Despite the existence of several lines of evidence on the anticancer potential of Ag NPs, reports regarding histidine-capped Ag NPs are deficient, as L-HAgNPs were mostly used for electrochemical studies. Hence, this work focuses on the fabrication of AgNPs using L-histidine as a reducing agent and evaluates the anticancer potential against SiHa cells.

## 2. Materials and Methods

### 2.1. Reagents Required

Silver nitrate, Sodium hydroxide, Penicillin, streptomycin, trypsin, and EDTA were procured from Himedia laboratories (Mumbai, India), while L-histidine (C_6_H_9_N_3_O_2_) was obtained from Merck Specialties Pvt Ltd., Mumbai, India. SiHa cells were purchased from National Centre for Cell Science (NCCS), Pune, India. Cell culture medium was procured from Sigma Aldrich Pvt Ltd., Bangalore, India.

### 2.2. Synthesis of L-HAgNPs

L-histidine and silver nitrate were prepared in 3 mM concentration in 30 mL of double-distilled water (DD). 1 M NaOH was used as a stabilizing agent. L-histidine and AgNO_3_ were mixed in the ratio 5:1 in two necked glass stopper flasks and stirred for 10 min, followed by the addition of 1 M NaOH until the pH reached 10. The reaction mixture was stirred continuously at 65 °C with a stirring speed of 450 rpm for 4 h until the color changes to brown confirming the formation of Ag nanoparticles (AgNPs) [20].

### 2.3. Characterization of Synthesized L-Histidine-Capped AgNPs (L-HAgNPs)

Formation of L–HAgNPs was confirmed by the color transition from pale yellow to brown color, which was further substantiated by UV–Visible spectral analysis with a scan range of 200–800 nm (Jasco International Co., Ltd., Tokyo, Japan). Fourier-transform infrared spectroscopy (FTIR) of freeze-dried samples was conducted in a PerkinElmer FTIR spectrophotometer (PerkinElmer, MA, USA) with the wavenumber range 4000–400 cm^−1^ at a resolution of 1 cm^−1^. The particle size and surface charge were analyzed by dynamic light scattering and zeta potential analyzer, while surface morphology and topology of the nanoparticles were characterized by AFM (Park System, Suwon, Korea), and scanning electron microscope (SEM) (Zeiss Supra-40 analytical SEM, Oberkochen Germany). The crystalline nature of synthesized Nanoparticles was assessed by X-ray Powder Diffraction (XRD) analysis (Rigaku Minifex-II, Tokyo, Japan) with Cu-Kα radiation of wavelength 1.54 Å

### 2.4. In Vitro Cytotoxicity of L-HAgNPs

SiHa cells were maintained in DMEM medium supplemented with fetal bovine serum (10%) and antibiotics (2%) at 37 °C in a CO_2_ incubator (5%) with 95% humidity. The passage number of cells used for experimentation was below 15. Briefly, 5 × 10^3^ cells per well were seeded in 96 well microtiter plate and exposed to different doses of L-HAgNPs (20–100 µg/mL) with DMSO as negative control and cisplatin as a positive control for 24 h, followed by incubation with 3-(4,5-Dimethylthiazol-2-yl (MTT) (5 mg/mL) for 3 h at a physiological temperature under dark condition [21]. Formed purple-colored crystals were dissolved in Dimethyl sulfoxide (DMSO) and the color intensity was measured at 570 nm using an ELISA plate reader (Bio-Rad, iMark, Hercules, CA, USA), and the percentage of cytotoxicity was calculated. IC 50 was calculated using probit software analysis.

### 2.5. Effect of L-HAgNPs on Intracellular ROS Level

Intracellular reactive oxygen species (ROS) levels in L-HAgNPs treated cells were assessed by using nonspecific esterase dichlorofluorescein diacetate, which, on oxidation with cellular peroxides, form green fluorescing compound 2,7-dichlorofluorescein. Cells after exposure to L-HAgNPs and cisplatin at its IC_50_ concentration were incubated with H2DCFDA at 37 °C for 30 min. Fluorescence intensity was measured by using a microplate reader with λEx/λEm of 485 nm/535 nm [22], and the cells were also visualized under a fluorescent microscope.

### 2.6. Effect of L-HAgNPs on Transmembrane Potential of Mitochondria (MMP/ΔΨm)

Alteration in the transmembrane potential of mitochondria was assessed using fluorescent dye JC-1, which emits orange-red fluorescence in the healthy cells and green fluorescence on membrane depolarisation. SiHa cells cultured on coverslips were exposed to cisplatin and L-HAgNPs for 12 h, followed by JC-1 staining (10 µM/mL) for 30 min in dark 37 °C. Excess dye was removed by PBS wash and the cells were viewed under a fluorescent microscope at absorption/emission maxima of 485/585 nm to observe the alteration in MMP.

### 2.7. Assessment of Apoptotic Effect of L-HAgNPs in SiHa Cells

#### 2.7.1. Acridine Orange (AO) and Ethidium Bromide (EB) Dual Staining

Morphological changes in the cells due to apoptosis were evaluated based on the methodology of Spector et al. (1998) [23]. SiHa cells seeded (0.3 × 10^6^ cells/well) in a six-well plate were exposed to L-HAgNPs and cisplatin at its IC_50_ concentration for 24 h, followed by ice-cold PBS (pH 7.4) wash. Cells were then treated with an equal proportion of AO/EtBr (1 mg/mL) for 10 min, and excess dye was removed and visualized under a fluorescent microscope (Carl Zeiss, Axioscope 2 plus) with blue and green filter. In each sample, the percentage of apoptotic and necrotic cells were quantified by examination of around 300 cells for normal, apoptotic, and necrotic morphology.

#### 2.7.2. Hoechst 33528 Staining

Overnight seeded cells exposed to L-HAgNPs were harvested and stained with Hoechst 33,258 dye (1 mg/mL) [24]. The cells with normal and abnormal nuclei were visualized under a fluorescent microscope using a blue filter (377–355 nm). Over 300 cells were counted, and the percentage of cells with normal and abnormal nuclei was calculated.

#### 2.7.3. Comet Assay

To assess the DNA damage induced by L-HAgNPs, a comet assay was performed based on the methodology of Tice et al. (2000) [25], with slight modifications. Cells exposed to L-HAgNPs were harvested, mixed with 1% low melting agarose, and coated on slides, followed by layering of normal melting agarose. Slides were incubated lysis buffer (TBE, pH 8.4, containing 2.5% Sodium dodecyl sulfate (SDS)) for 10 min and then subjected to electrophoresis in TBE buffer for 10 min without SDS at 2 V/cm and 80 mA, respectively. Slides were subjected to EtBr staining, washed, covered with coverslips, and stored at 4 °C until visualized under a fluorescent microscope with excitation/emission wavelength of 515/560 nm. Tail length, olive tail moments were measured using Komet 5.5 image analysis software (Komet image analysis version 5.5, Oxford instruments, Abingdon, UK).

#### 2.7.4. Annexin V-Cy3 Staining

Annexin V-Cy3 dual staining was carried out to differentiate the live (fluoresces green), necrotic (fluoresces red), and apoptotic cells (exhibits both red and green fluorescence) A549 cells (0.3 × 10^6^ cells/well) grown in coverslip were exposed to L-HAgNPs and Cislatin at its IC_50_ concentration for 12 h, followed by a wash with PBS and annexin binding buffer (1X). Cells were stained with dual staining solution (annexin V-cy3 and 6-CFDA) for 10 min in dark and visualized under a fluorescent microscope. Around 300 cells from each group were counted, and the percentage of viable and dead (apoptotic and necrotic) cells were calculated.

### 2.8. Statistics Analysis

Results obtained from three independent experiments were represented as Mean ± SD and analyzed using one-way analysis of variance (ANOVA), and the average value was compared by Duncan’s multiple comparison tests (SPSS 17.0).

## 3. Results

### 3.1. Formation of AgNPs

**Figure 1:**
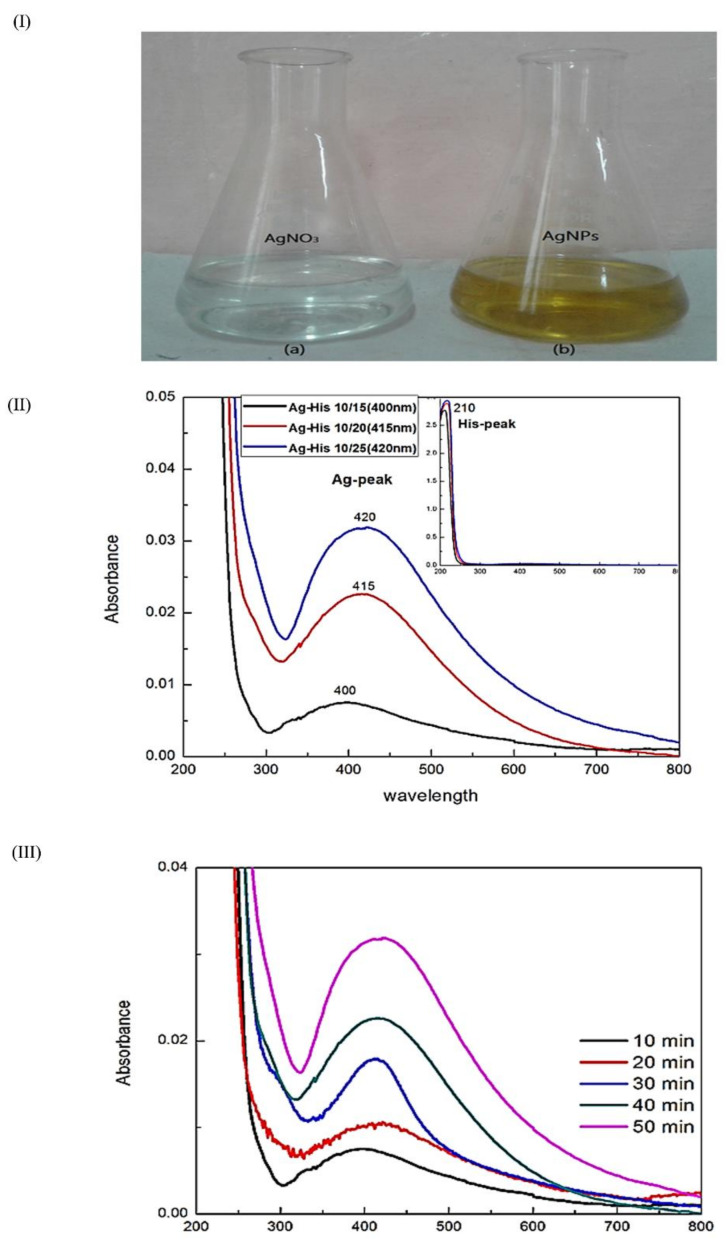
(**I**) Color transition illustrating the formation of AgNPs; (**II**) UV–Visible spectra of AgNPs synthesized using various molar ratios of AgNO_3_ and L-histidine; (**III**) UV–Visible spectrum illustrating the optimization of time for the synthesis of AgNPs.

Reduction of Ag (NO_3_)_2_ by L-histidine was identified by the color transition from colorless to brown color, which was further confirmed by UV–Visible spectral analysis (Figure 1I). A sharp absorption peak was observed between 400 and 420 nm. The absorption peak of AgNPs synthesized using various molar ratios of AgNO_3_ with L-histidine (1:1, 1:2, and 1:2.5) showed peaks at 400, 415, and 420 nm, respectively (Figure 1II). Figure 1III showed the absorption spectrum of Ag NPs at various time intervals during its synthesis illustrating the time-dependent reduction of AgNO_3_ to AgNPs with maximum reduction at 50 min.

### 3.2. Particle Size Analysis by DLS

**Figure 2:**
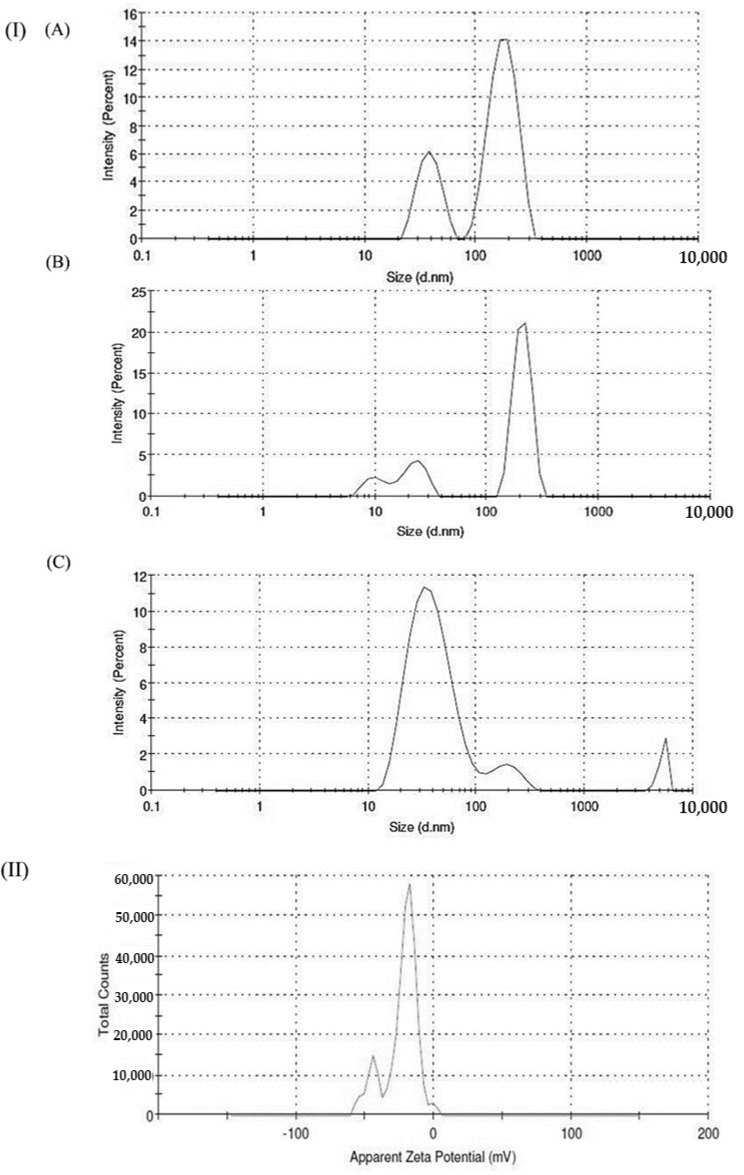
(**I**) DLS spectrum illustrating the particle size of AgNPs synthesized using various molar ratios of precursor and (**A**) AgNO_3_: L-histidine 1:1, (**B**) AgNO_3_: L-histidine 1:2, and (**C**) AgNO_3_: L-histidine 1:2.5; (**II**) zeta potential analysis of AgNPs.

The size of synthesized AgNPs was determined using particles size distribution analysis by measuring the random changes in the DLS. It was found that the average size of L-HAgNPs was observed to be 445.3 ± 36.44, 145.5 ± 47.99, and 47.43 ± 19.83 nm, respectively for the precursor AgNO_3_ with L-histidine in the ratio 1:1, 1:2, and 1:2.5 (Figure 2I(A–C)). The higher the ratio of AgNO_3_ with L-histidine (1:2.5) used for the fabrication of AgNPs, the lower the particle size. The polydispersity index was found to be 0.5 ± 0.2, which shows a monodisperse distribution for the particles. The zeta potential value of L-HAgNPs synthesized using a precursor ratio of 1:2.5 was observed to be −20.5 ± 0.95 mV (Figure 2II).

### 3.3. X-ray Diffraction Studies of Silver Nanoparticles

**Figure 3:**
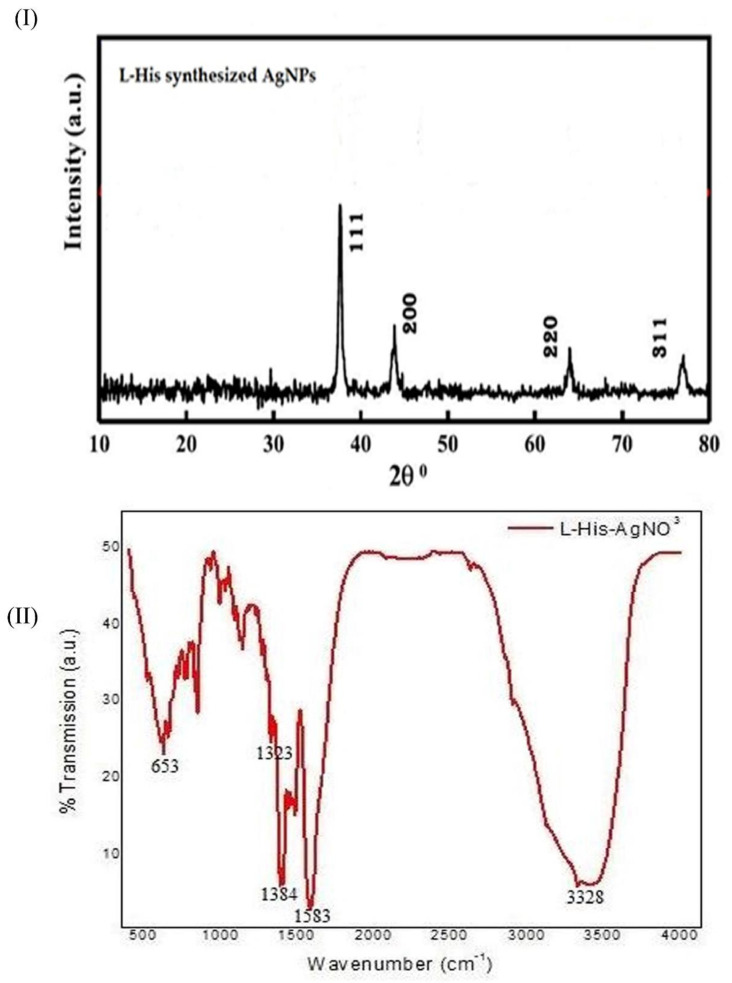
(**I**) X-ray diffraction pattern of L-HAgNPs; (**II**) Fourier-transform infrared spectra of L-HAgNPs.

The nature and crystalline structure of L-HAgNPs were assessed by X-ray diffraction analysis, and results are depicted in Figure 3I. XRD analysis of L-HAgNPs showed diffraction peaks at 38.1°, 44.3°, 64.7°, 77.1° assigned to the planes (111), (200), (220), (311) facet, corresponding to JCPDS No:01-087-0597, thereby confirming face-centered cubic crystalline structure (Figure 3I). The mean crystal size of L-HAgNPs was calculated using Debye–Scherrer’s formulae as 9.76 nm.

### 3.4. Functional Group Analysis

The functional group involved in the chemical interaction between L-histidine and AgNPs was evaluated using FT-IR and illustrated in Figure 3II. Visible bands were observed at 653 cm^−1^, 1323 cm^−1^, 1384 cm^−1^, 1583 cm^−1^, and 3328 cm^−1^, which were assigned to C-H group bending vibration, C-N stretching vibration of imidazole group and –COOH group, N-H bending, O-H group of H_2_O depicting the presence of histidine in L-HAgNPs. The characteristic peak at 1563 cm^−1^ and 1460 cm^−1^ depicts the stretching frequencies of carboxylate (COO^-^), and the peak at 3328 cm-1 corresponds to the stretching vibration of the N-H group, which is analogous to aminocarboxylates and carboxylates present in L-histidine (Figure 3II).

### 3.5. Size and Morphology Analysis of L-HAgNPs by SEM, HR-TEM, and AFM

**Figure 4:**
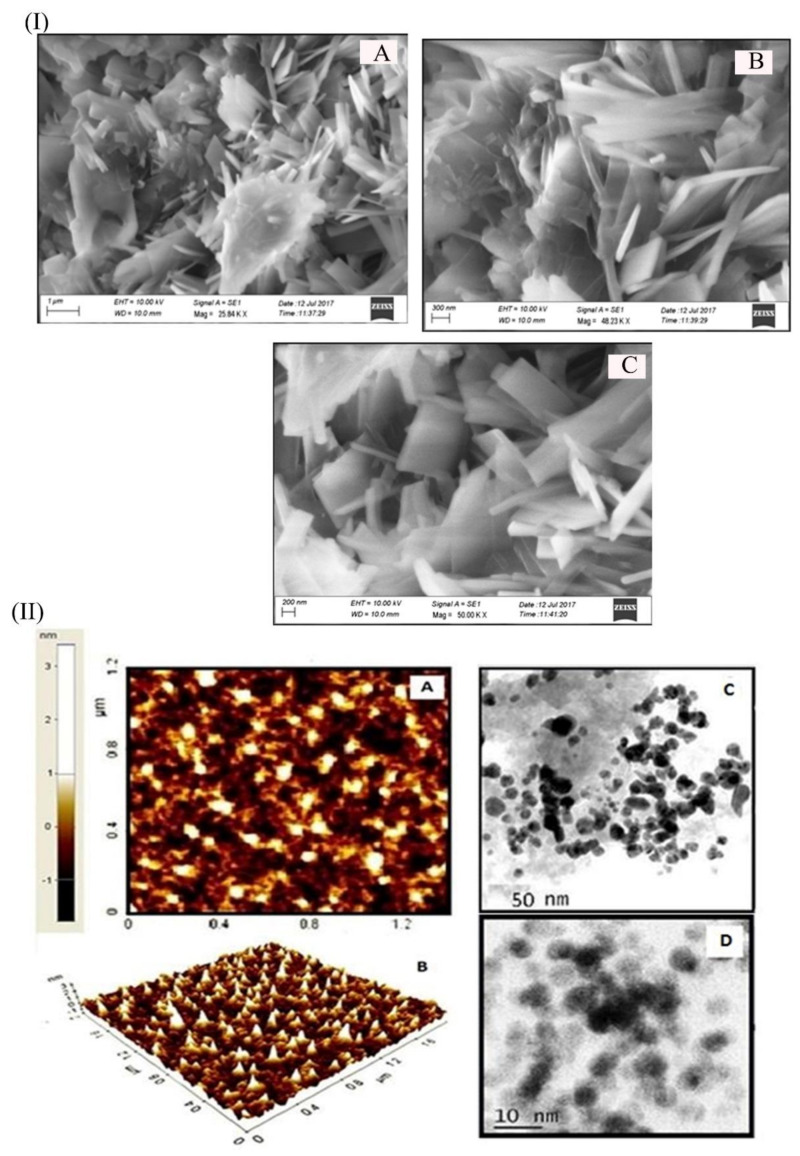
Morphological assessment of L-HAgNPs by (**I**) SEM images at different magnifications: (**A**) 1 µM, (**B**) 300 nm, and (**C**) 200 nm; (**II**) AFM micrograph: (**A**) 2D image, (**B**) 3D image, and (**C**,**D**) TEM micrographs.

The size, shape, and surface morphology of synthesized nanoparticles were analyzed through SEM, and results are illustrated in Figure 4I(A–C). Results showed the presence of needle-shaped crystalline structure in L-HAgNPs in different magnification. The 2D and 3D images of AFM results revealed the presence of irregular spherical shaped nanoparticles, and the average height of L-HAgNPs was observed to be between scale 10 to 16 nm (Figure 4II(A,B)). L-histidine acts as a capping agent for AgNPs, thereby preventing the agglomeration of AgNPs. Further, the morphology of AgNPs was assessed by HR-TEM, and the results depicted the presence of dark-colored uniform-sized AgNPs capped by light-colored L-His material with an average size of 20 nm (Figure 4II(C,D)).

### 3.6. Anticancer Potential of L-HAgNPs against SiHa Cells

#### 3.6.1. Dose-Dependent Cytotoxicity Effect of L-HAgNPs in SiHa Cells

**Figure 5:**
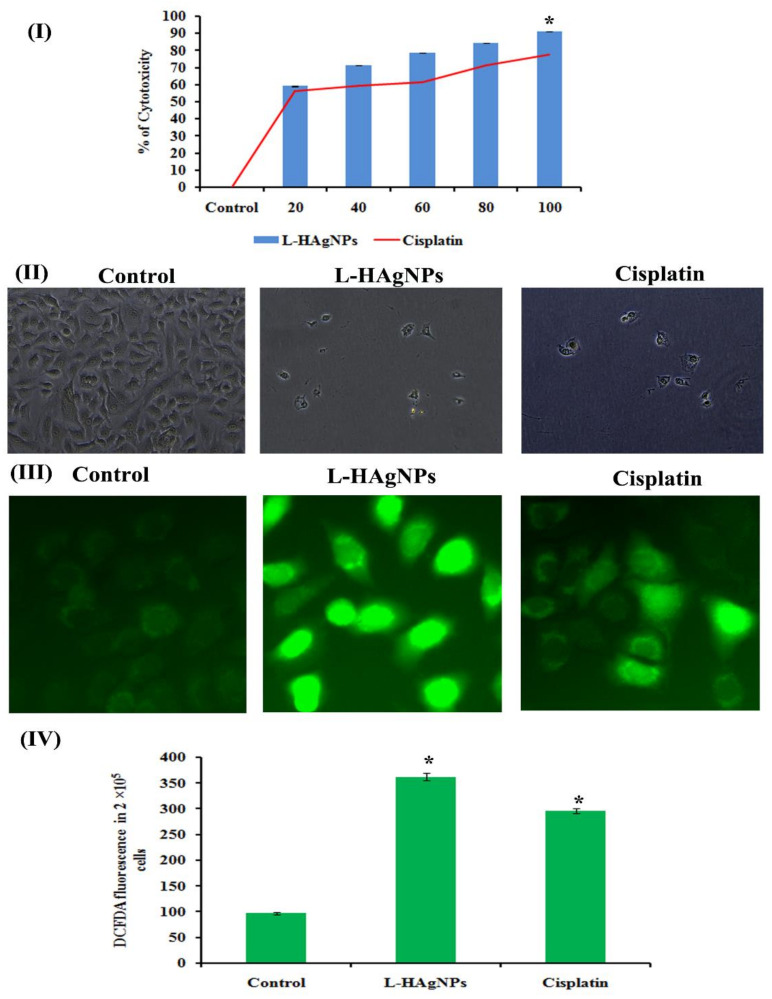
(**I**) Antiproliferative effect of L-HAgNPs against SiHa cells; (**II**) phase-contrast microscopic images to assess structural changes in cells treated with L-HAgNPs; (**III**) fluorescent images illustrating intracellular ROS level; (**IV**) bar graph depicting quantitative measurement of green fluorescent intensity proportional to ROS level. * *p* < 0.05 denotes statistical significance between control vs. treated groups.

Antiproliferative effect of L-HAgNPs against SiHa cells was assessed using MTT assay; L-HAgNPs showed a dose-dependent cytotoxic effect (20–100 µg/mL), with IC_50_ value of 18.25 ± 0.36 µg/mL, similar to positive control cisplatin, whose IC_50_ value was observed to be 19.15 ± 0.16 µg/mL (Figure 5I). Phase-contrast microscopic analysis revealed the presence of intact cells with normal cervical epithelial morphology in vehicle control cells, while L-HAgNPs and cisplatin-treated cells showed reduced colonies with shrunken morphology, disrupted cell membrane, and fragmented nuclei (Figure 5II).

#### 3.6.2. Effect of L-HAgNPs on the Intracellular ROS Level of Treated SiHa Cells

To elucidate the mechanism behind apoptosis in L-HAgNPs treated SiHa cells, endogenous ROS level was measured, and the results are illustrated in Figure 5III,IV. Fluorescent microscopic studies revealed the presence of intense green fluorescent cells in L-HAgNPs treated and cisplatin-treated groups, while control cells showed less intense fluorescence. Quantitative analysis revealed fourfold enhanced fluorescent intensity (408.33 ± 6.83 a.u.) in L-HAgNPs when compared with control cells, while cisplatin-treated cells showed threefold increase in fluorescent intensity (296.33 ± 4.926 a.u), depicting the fact that L-HAgNPs enhanced the intracellular ROS level the key mediator of apoptosis.

#### 3.6.3. Effect of L-HAgNPs on the Transmembrane Potential of Treated SiHa Cells

**Figure 6:**
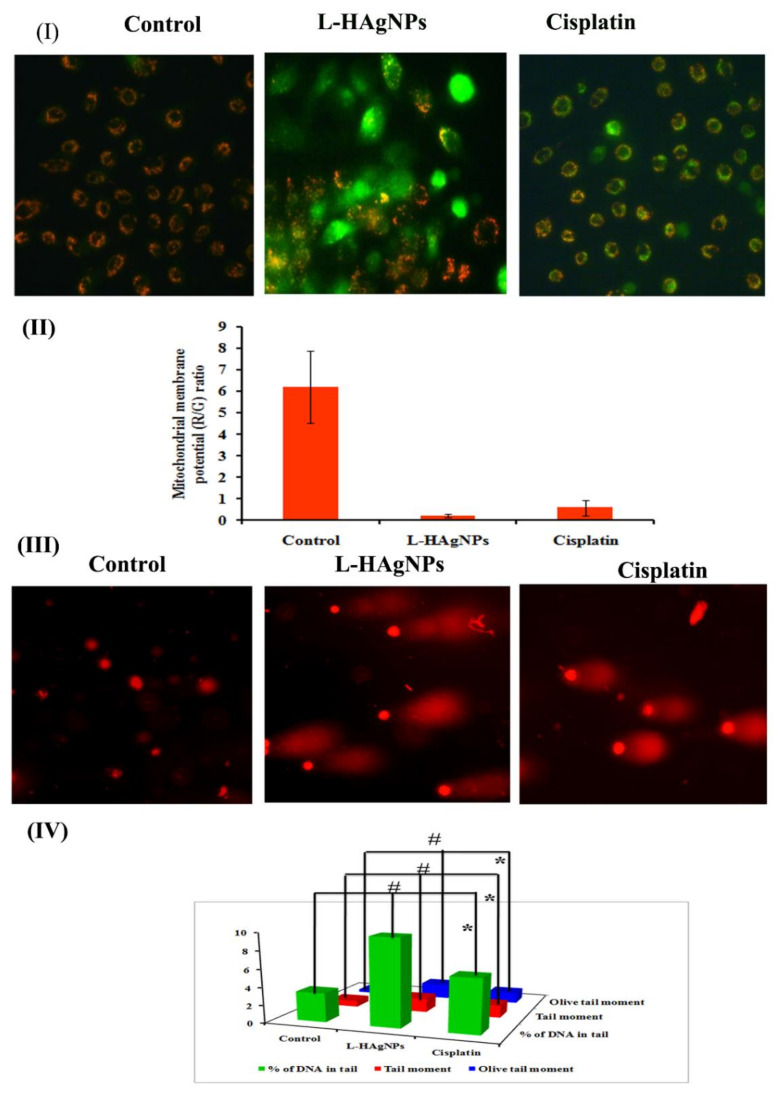
(**I**) Fluorescent micrograph revealing transmembrane potential of cells treated with L-HAg NPs; (**II**) bar graph representing quantification of MMP; (**III**) fluorescent microscopic images revealing DNA damage induced by L-HAgNPs; (**IV**) quantitative measurement of degree of DNA damage. Data are expressed as mean ± SD of triplicate assays. Images were captured at 20× magnification, respectively. # and * *p* < 0.0001 denotes statistical significance between control vs. L-HAgNPs treated groups and control vs. cisplatin-treated groups.

To assess the impact of L-HAgNPs on mitochondrial function in SiHa cells, its transmembrane potential was assessed using fluorescent dye JC-1. Figure 6I revealed the presence of red fluorescence in vehicle control cells, while in L-HAgNPs and cisplatin-treated cells showed disappearance of red fluorescence and appearance of green fluorescence. Quantification of red-to-green fluorescent ratio revealed that L-HAgNPs and cisplatin-treated cells showed a sixfold reduction when compared with vehicle control (Figure 6II).

#### 3.6.4. Assessment of DNA Damage Induced in L-HAgNPs by Comet Assay

To evaluate the impact of L-HAgNPs on DNA, single-cell gel electrophoresis was carried out, and the results are shown in Figure 6III,IV. Vehicle control groups revealed cells with intact nuclei, which appear round (Class 0), while L-HAgNPs and cisplatin-treated groups showed the presence of nuclei with fragmented DNA, which migrates faster as tail, giving the appearance of comet (Classes 2 and 3) (Figure 6III). Quantification of tail moment and percentage of DNA in the tail showed a threefold increase in tail moment (1.56 ± 0.013), olive tail moment (1.721 ± 0.001), and % of DNA in tail (97.0 ± 0.08) in L-HAgNP-treated groups when compared with vehicle control.

#### 3.6.5. Effect of L-HAgNPs on Apoptosis by AO/EtBr Dual Staining

**Figure 7:**
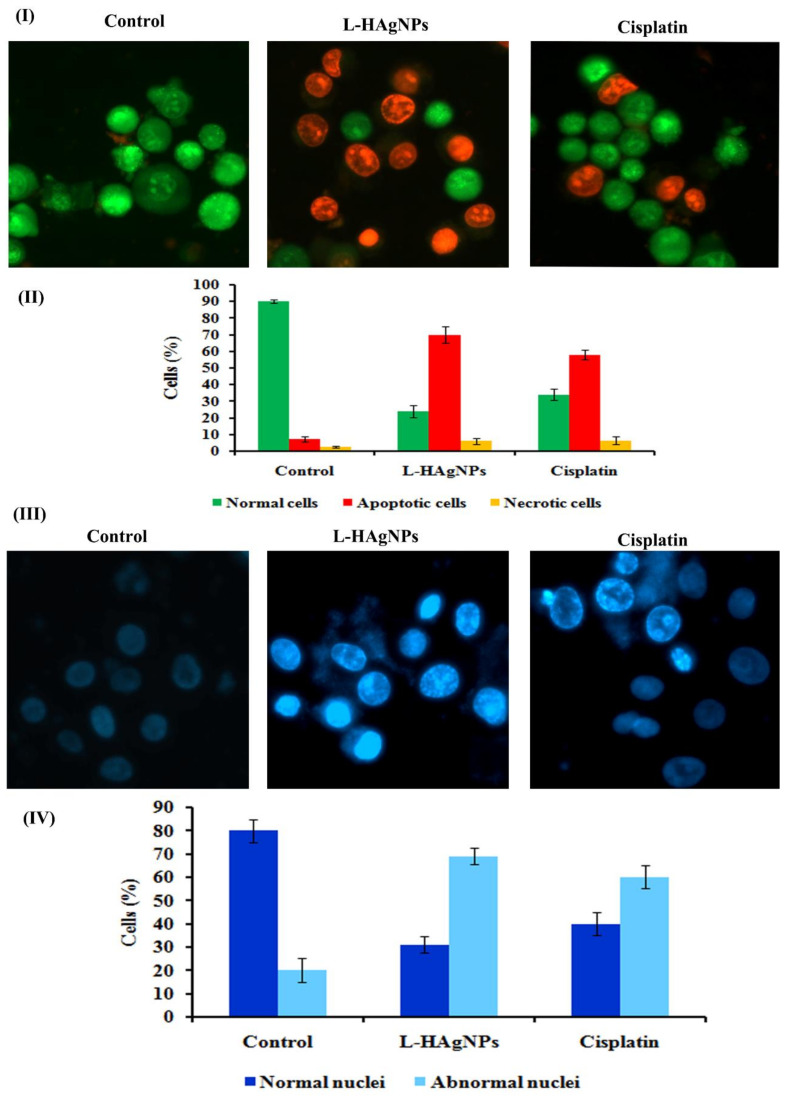
Assessment of apoptotic effect of L-HAgNPs against cervical cancer cells: (**I**) fluorescent image of AO/EtBr dual staining; (**II**) quantification of apoptotic and necrotic cells; (**III**) images representing nuclear damage by Hoechst 33,528 staining; (**IV**) bar graph illustration percentage of cells with normal and apoptotic nuclei.

To determine the extent of apoptosis induced by L-HAgNPs in SiHa cells, AO/EtBr double staining was carried out. Figure 7I showed the presence of intact green fluorescent cells with organized structure in vehicle control, while L-HAgNP- and cisplatin-treated groups illustrated the presence of both granular yellow/green stained cells and orange red fluorescence cells. Quantification of normal, apoptotic and necrotic cells showed 90 ± 1% viable intact cells, 7.33 ± 1.52% apoptotic cells, and 2.667 ± 0.57% necrotic cells in vehicle control. L-HAgNPs treated groups exhibited 24 ± 3.06% viable cells, 70 ± 5% apoptotic cells, and 6 ± 1.73% necrosis. Similar results were observed in cisplatin treated cells (34 ± 6.33% viable cells: 58 ± 2.64% apoptosis and 6.33 ± 2.30% necrosis) (Figure 7II). 

#### 3.6.6. Assessment of Nuclear Damage by Hoechst 33528 Nuclear Staining

To assess whether apoptosis is due to nuclear damage Hoechst 33,528 staining was carried out, and the results are illustrated in Figure 7III,IV. Control cells showed presence of cells with intact nucleus and cytoplasm exhibiting light blue fluorescence, while L-HAgNP-treated cells revealed the presence of intense blue-fluorescent cells, indicating the presence of apoptotic nuclei (Figure 7III). Percentage of apoptotic and necrotic cells were assessed, and the results indicated that L-HAgNP-treated cells showed 31 ± 3.6% normal nuclei and 69 ± 3.6% apoptotic nuclei when compared with vehicle control (80 ± 5% normal nuclei and 20 ± 5% abnormal nuclei). Cisplatin-treated cells showed 40 ± 5% cells, with intact nuclei and 60 ± 5.2% damaged nuclei (Figure 7IV).

#### 3.6.7. Assessment of Apoptotic Effect of L-HAgNPs by Annexin V-Cy3 Dual Staining

**Figure 8:**
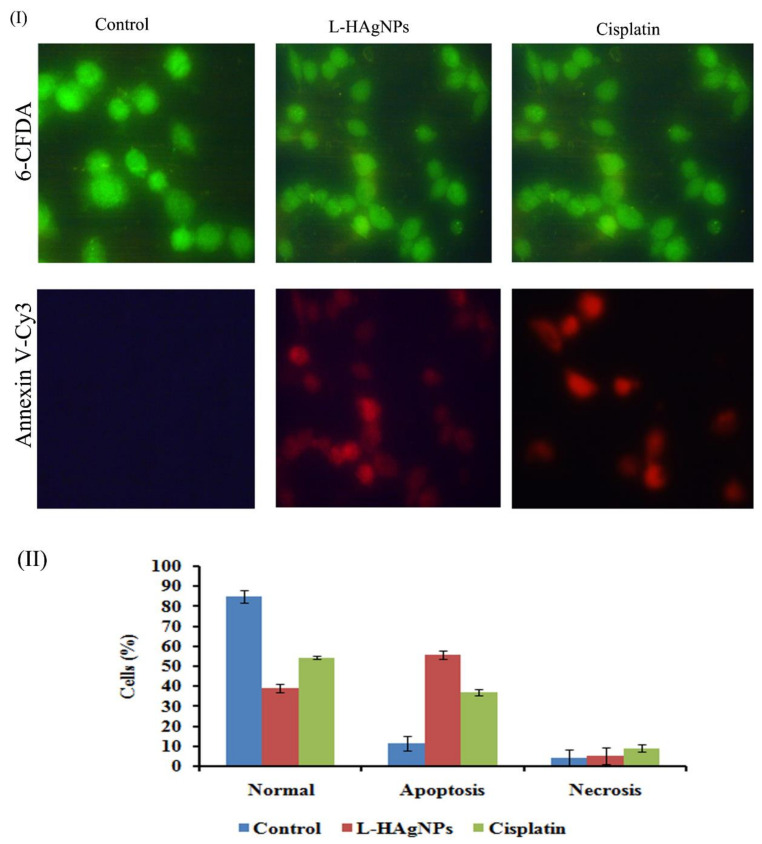
Assessment of L-HAgNPs induced apoptosis in cervical carcinoma cells (SiHa) by annexin-Cy3 (red) and 6-CFDA (green) dual staining (**I**) Fluorescence microscopic image representing apoptotic (green) and healthy cells (red); (**II**) percentage of apoptotic cells.

To substantiate the apoptosis mediated cell death in L-HAgNP-treated groups annexin V-Cy3 dual staining was performed, and the results are shown in Figure 8. Results revealed the presence of green-fluorescent cells stained with annexinV-Cy3, while cells treated with L-HAgNPs and cisplatin exhibited both red and green fluorescence, indicating apoptosis mediated cell death (Figure 8I). Quantification of cells showed that L-HAgNP-treated groups exhibited 55.66 ± 2.08% apoptotic cells, 5.33 ± 1.52% necrotic cells and 39 ± 3.6% normal cells when compared with control, which showed 84.66 ± 4% healthy cells. Cisplatin-treated groups showed 54.33 ± 4.04% healthy cells, 36.66 ± 4.16% apoptotic cells and 9 ± 1.73% necrotic cells (Figure 8II).

## 4. Discussion

Traditional cancer therapeutics involves combinatorial therapy of chemo/Radio/surgery, which has certain limitations such as poor bioavailability, severe side effects due to nonspecific drug delivery, multidrug resistance, and toxicity [26]. Advancement of nanotherapeutics in cancer therapy helped in overcoming limitations such as lack of solubility, specificity, and multidrug resistance. Among the noble metal nanoparticles, AgNPs have been extensively studied for its antitumor potential against various cancer cell lines. Tunable size, plasmonic nature, specificity, and eco-friendly nature of AgNPs makes it a promising theranostic agent for cancer therapy [27,28,29]. Despite the existence of several lines of evidence on anticancer potential of AgNPs, reports on anticancer potential of Lhistidine-capped AgNPs are deficient; hence, this study focused on synthesizing L-histidine-capped AgNPs for cervical cancer therapy.

Fabrication of L-HAgNPs was identified by the color transition from colorless to brown color and further confirmed by the UV–Visible absorption spectrum between 400 and 420 nm, as previously reported by Xu et al. (2020) [30]. Particle size analysis revealed that increased ratio of precursor AgNO3 and L-histidine decreased the size of synthesized nanoparticles, with the least particle size of 47.43 ± 19.83 nm for the precursor ratio AgNO_3_: L-histidine (1:2.5). Polydispersity index was observed to be 0.5 ± 0.2, indicating uniform dispersion of nanoparticles, as reported by Danaei et al. (2018) [31]. As the particle size of the fabricated nanoparticle is less than 100 nm, these nanoparticles can efficiently be internalized into the cells in accordance to the report of Foroozandeh and Aziz (2018) [32]. Zeta potential value of L-HAgNPs synthesized was observed to be −20.5 ± 0.95 mV. Negative charge of L-HAgNPs may be derived from COO- group of histidine, and the strong electronegative charge prevents agglomeration of nanoparticles due to electrostatic force of repulsion. The negative zeta potential of L-HAgNPs also promotes the interaction with positively charged site, i.e., transmembrane protein of cell membrane through electrostatic force of attraction, leading to localized neutralization, which favors endocytosis-mediated cellular uptake [33]. XRD analysis of L-HAgNPs revealed face centered cubic crystalline structure, as reported by Iswarya et al. (2017) [17]. FTIR analysis revealed that presence of signals for primary amino group (3328 cm^−1^) and carboxyl group (1460 cm^−1^), which is analogous to aminocarboxylates and carboxyl groups present in L-histidine, as reported by Iswarya et al. (2017) [17]. In this study, signals for CH-COO- (1410–1430 cm^−1^) was not detected, indicating the fact that AgNPs colloids are covered fully by imdiazole ring of L-histidine while carboxyl group is exposed outwards with no contact with AgNPs as per the report Liu et al. (2010) [34]. The morphology of L-HAgNPs was probed using SEM, which showed the presence of clusters of needle-shaped structure clearly visible at 200 nm magnification. Negative charge of L-HAgNPs facilitates the absorption of AgNPs on the 3-Aminopropyl)triethoxysilane (APTES) modified surface via electrostatic interaction for AFM observation. AFM results showed the presence of uniform distribution spherical-shaped structure with average height of 12 ± 4 nm, which is in accordance to the results of TEM imaging [14].

Anticancer potential of L-HAgNPs against cervical cancer was assessed using SiHa cells the most widely used model system. MTT assay exhibited an enhanced dose-dependent reduction in proliferation rate when compared with platinum-based drug cisplatin. In addition, morphological analysis of L-HAgNP-treated groups exhibited a reduction in cell density, adhesion capacity, loss of typical cell shape with shrunken morphology the apoptotic features, suggesting the fact that cytotoxic effect of AgNPs might be due to antineoplastic nature as observed in previous reports [18,19]. Most chemotherapeutic drugs act by elevating intracellular ROS levels disproportionally, leading to mitochondrial dysfunction, thereby activating intrinsic apoptotic pathways. Therefore, the assessment of intracellular ROS level and mitochondrial membrane potential acts as an indicator of healthy cells [34]. In this study, the intense green fluorescent intensity in the L-HAgNP-treated group depicts an increase in the intracellular ROS level, the key factor leading to mitochondrial dysfunction ultimately activating apoptotic cascade [35,36]. To evaluate the mitochondrial damage, MMP was assessed using JC-1 staining. Vehicle control cells showed enhanced red fluorescence due to the formation of J-aggregates, while L-HAgNP-treated cells exhibited enhanced green fluorescence due to loss of electrochemical potential, which might be due to elevated ROS level, leading to alteration in the mitochondrial membrane permeability as observed in the reports of Yuan et al. (2018) [19]. Recent studies reported that nanoparticle-treated cancer cells exhibited enhanced ROS bursts, which led to oxidative modification of biomolecules such as protein oxidation, DNA fragmentation, and lipid peroxidation, leading to cell death [37]. To verify whether apoptosis is caused due to ROS-induced DNA damage, induced by elevated ROS levels, a comet assay was carried out, which is a widely used method to assess single-strand and double-strand breaks in DNA [38]. Results of this study revealed that L-HAgNP-treated cells showed a significant increase in the olive tail moment, tail length, and around 90% of DNA in the tail when compared with vehicle control, confirming the fact that L-HAgNPs induced significant DNA damage, which acts as one of the key factors for apoptosis. To elucidate the mechanism of cell death, AO/EtBr dual staining was carried out, and the results showed the presence of both green and orange fluorescing cells in the L-HAgNP-treated group, depicting both apoptosis- and necrosis-mediated cell death. To detect whether the mechanism of cell death is due to DNA fragmentation, Hoechst 33,528 staining was carried out, which showed the presence of intense blue fluorescent cells with condensed and fragmented nuclei in L-HAgNP-treated cells, coinciding with previous reports on AgNP-treated cervical cancer cells [39]. Annexin V/6CFDA dual staining widely used to segregate viable cells from nonviable cells substantiated the fact that L-HAgNPs induced apoptosis-mediated cell death rather than necrosis. Collectively, the findings of this study reveal that the fabricated L-HAgNPs induce ROS-mediated mitochondrial membrane alteration, thereby activation intrinsic apoptotic pathway leading to cell death, concluding that the L-HAgNPs can effectively mitigate cervical carcinoma.

## 5. Conclusions

This study focused on the synthesis of water-dispersible, ecofriendly L-histidine functionalized AgNPs by a single-step one-pot approach. Fabricated L-HAgNPs in different ratios of precursors exhibited characteristic SPR peaks within a wavelength range of 400–450 nm, corresponding to AgNPs. FT-IR spectrum revealed that L-histidine acts as a reducing and capping agent for L-HAgNPs. SEM topographical images of L-HAgNPs revealed crystalline needle-shaped structure, while DLS revealed the size less than 50 nm suitable for cellular internalization. The anticancer potential of L-HAgNPs revealed the antineoplastic effect in a concentration-dependent manner. Elucidation of the anticancer mechanism illustrated loss of MMP due to increased ROS level, thereby activating apoptotic pathways and inducing DNA damage, ultimately leading to cell death. Overall, the results reveal that L-HAgNPs is a biocompatible potent candidate for cancer therapy.

## Data Availability

Not applicable.
